# Upregulation of 5-Hydroxytryptamine Receptor Signaling in Coronary Arteries after Organ Culture

**DOI:** 10.1371/journal.pone.0107128

**Published:** 2014-09-09

**Authors:** Chun-Yu Deng, Hui Yang, Su-Juan Kuang, Fang Rao, Yu-Mei Xue, Zhi-Ling Zhou, Xiao-Ying Liu, Zhi-Xin Shan, Xiao-Hong Li, Qiu-Xiong Lin, Shu-Lin Wu, Xi-Yong Yu

**Affiliations:** 1 Medical Research Center of Guangdong General Hospital, Guangzhou, P.R. China; 2 Guangdong Provincial Cardiovascular Institute, Guangdong Academy of Medical Sciences, Guangzhou, P.R. China; Universidade Federal do Rio de Janeiro, Brazil

## Abstract

**Background:**

5-Hydroxytryptamine (5-HT) is a powerful constrictor of coronary arteries and is considered to be involved in the pathophysiological mechanisms of coronary-artery spasm. However, the mechanism of enhancement of coronary-artery constriction to 5-HT during the development of coronary artery disease remains to be elucidated. Organ culture of intact blood-vessel segments has been suggested as a model for the phenotypic changes of smooth muscle cells in cardiovascular disease.

**Methodology/Principal Findings:**

We wished to characterize 5-HT receptor-induced vasoconstriction and quantify expression of 5-HT receptor signaling in cultured rat coronary arteries. Cumulative application of 5-HT produced a concentration-dependent vasoconstriction in fresh and 24 h-cultured rat coronary arteries without endothelia. 5-HT induced greater constriction in cultured coronary arteries than in fresh coronary arteries. U46619- and CaCl_2_-induced constriction in the two groups was comparable. 5-HT stimulates the 5-HT_2A_ receptor and cascade of phospholipase C to induce coronary vasoconstriction. Calcium influx through L-type calcium channels and non-L-type calcium channels contributed to the coronary-artery constrictions induced by 5-HT. The contractions mediated by non-L-type calcium channels were significantly enhanced in cultured coronary arteries compared with fresh coronary arteries. The vasoconstriction induced by thapsigargin was also augmented in cultured coronary arteries. The decrease in Orai1 expression significantly inhibited 5-HT-evoked entry of Ca^2+^ in coronary artery cells. Expression of the 5-HT_2A_ receptor, Orai1 and STIM1 were augmented in cultured coronary arteries compared with fresh coronary arteries.

**Conclusions:**

An increased contraction in response to 5-HT was mediated by the upregulation of 5-HT_2A_ receptors and downstream signaling in cultured coronary arteries.

## Introduction

5-Hydroxytryptamine (5-HT) is an important signaling molecule in regulation of the cardiovascular system. It is stored primarily in platelets and is released into plasma if activated at injury sites [1] and in patients with coronary artery disease (CAD) [2]. 5-HT can produce harmful acute and chronic effects. It promotes platelet aggregation, vasoconstriction, and proliferation of vascular smooth muscle cells (VSMCs) [3]. Hence, high levels of 5-HT in plasma are associated with accelerated cardiovascular events [4].

5-HT is a powerful constrictor of coronary arteries in several species (including humans). 5-HT is considered to be involved in the pathophysiological mechanisms of coronary-artery spasm. This results in disturbances in the tone of coronary arteries, blood flow and, subsequently, a lack of supply of oxygen and nutrients to the heart.

5-HT receptors mediate its functions. 5-HT receptors are divided into seven subfamilies. The 5-HT_1A_, 5-HT_1B/1D_, 5-HT_2_ receptor family (5-HT_2A_ and 5-HT_2B_), 5-HT_3_, 5-HT_4_ and 5-HT_7_ receptors are found in cardiovascular tissues [5]. Only the 5-HT_3_ receptor family couples to ion channels. Other 5-HT receptor families are heptahelical receptors coupled to G proteins (Gs, Go, Gi, Gq/11) and have diverse effectors. 5-HT_2A_ and/or 5-HT_1_ receptor subtypes mediate 5-HT-induced vasoconstriction exclusively [6]. The 5-HT receptors mediating constriction in coronary arteries from different species are predominantly the 5-HT_2_ receptor and, to a lesser extent, 5-HT_1_ receptor [1], [7].

The vascular organ-cultured system has the distinct advantage of maintaining the differentiated contractile phenotype of smooth muscle cells because of better preservation of tissue architecture, cell-to-cell interactions, extracellular matrix, and cell morphology. Some studies have shown that organ culture of segments of intact blood vessels could be a model for the phenotypic changes that occur in VSMCs during the development of cardiovascular disease [8], [9]. Pathological conditions such as hypertension, atherosclerosis and diabetes are often associated with proliferation and differentiation of vascular smooth muscle cells and subsequent structural changes in the vascular wall [10]. Organ culture of rat coronary arteries induces upregulation of expression of contractile endothelin type-B receptors on VSMCs, thereby mimicking atherosclerosis and CAD [11], [12]. Organ culture also induces downregulation of the contractility of angiotensin-II receptors, thereby reflecting the phenotypic changes in human coronary arteries from patients with ischemic heart disease [13], [14]. Expression of 5-HT_1B/1D_ receptors is also upregulated after organ culture of rat cerebral arteries, which resembles the alterations in SMC function after subarachnoidal hemorrhage [15], [16], [17]. So far, this model of vascular disease has been applied to study the pharmacological characteristics and underlying molecular and cellular mechanism of vascular receptor alterations.

We investigated the vasoconstriction and expression of 5-HT receptors of rat coronary arteries after 24-h culture *in vitro*. Our results suggested that upregulation of expression of 5-HT_2A_ receptors and the downstream phospholipase C (PLC)/Orai1 signaling pathway could be responsible for an augmented contraction in response to 5-HT.

## Materials and Methods

The study protocol was approved by the Experimental Animal Ethics Committee of Guangdong General Hospital (Guangzhou, P.R. China).

### Tissue collection and organ culture [9]

Male Sprague–Dawley rats (220–280 g; 8–10 weeks) were used. Rats were housed under 12-h light–dark conditions with *ad libitum* access to water and food. Rats were euthanized with CO_2_ and decapitated. Hearts were removed immediately and chilled in ice-cold Kreb's solution (in mM): NaCl 119, KCl 4.7, CaCl_2_ 2.5, MgCl_2_ 1, NaHCO_3_ 25, KH_2_PO_4_ 1.2, and D-glucose 11.1. The left anterior descending coronary artery was excised from the myocardium. Adherent connective tissues were removed. The endothelium was also removed, and cut into two ringed segments (length, ≈2 mm, diameter, ≈250 µm.). Arterial segments were placed in wells containing Dulbecco's modified Eagle's medium (DMEM) supplemented with 10% fetal bovine serum (FBS), 100 U mL^−1^ penicillin, and 100 µg mL^−1^ streptomycin. Wells were incubated at 37°C in an atmosphere of 5% CO_2_ for 24 h, and thereafter mounted in myographs to record arterial tone. Other arterial segments used for real-time polymerase chain reaction (PCR) and western blotting experiments were frozen on dry ice and stored at −80°C.

### Measurement of vessel force

Measurement of vessel force was undertaken as described previously [18]. Each segment was mounted in a Multi Myograph system (Danish Myo Technology, Aarhus, Denmark) and changes in arterial tone recorded. Briefly, two tungsten wires (each with a diameter of 40 µm) were inserted through the lumen of the segment, and each wire fixed to the jaws of a myograph. The organ chamber was filled with 5 mL Krebs solution, which was bubbled constantly with a mixture of 95% O_2_ and 5% CO_2_ and maintained at 37°C. Each ring was stretched initially to 1.5 mN (optimal tension) and then allowed to stabilize at this baseline tone for 90 min before the start of each experiment. Each experiment was conducted using rings from different rats. The endothelium was removed mechanically by rubbing the luminal surface of the ring with small stainless-steel wire. 5-HT at 2 µM was used to precontract the vessels. Functional removal of the endothelium was verified if the relaxant effect of 1 µM acetylcholine was absent. In experiments using a high-K^+^ solution (60 mM), an equimolar amount of K^+^ replaced Na^+^ to retain constant ionic strength. We determined the contractile sensitivity to Ca^2+^. Rings were rinsed thrice using a Ca^2+^-free solution containing 50 µM Na_2_-EGTA, then incubated in Ca^2+^-free and 60 mM K^+^ solution before cumulative addition of CaCl_2_.

### Culture of VSMCs from rat coronary arteries

Coronary arteries were harvested immediately as mentioned above. Then, they were placed in ice-cold Kreb's solution (in mM): NaCl 119, KCl 4.7, CaCl_2_ 2.5, MgCl_2_ 1, NaHCO_3_ 25, KH_2_PO_4_ 1.2, and D-glucose 11.1. After removal of fat and connective tissue, coronary arteries were cut into small pieces (length, ≈0.2 mm) and placed in a 35-mm culture dish. Dulbecco's modified essential medium supplemented with 20% fetal bovine serum (FBS), 100 U mL^−1^ penicillin, and 100 µg mL^−1^ streptomycin was added. Cells were cultured at 37°C in an atmosphere of 5% CO_2_ for 3 days, after which the dish was replaced with fresh culture medium. SMCs migrated from the specimens in the vessel in 7–10 days. After reaching 80% confluence, cells were subcultured with 0.25% trypsin with 0.02% ethylenediamine tetra-acetic acid.

### *Ex vivo* lentivirus infection in coronary VSMCs

Before the day of infection, coronary VSMCs were seeded at 1×10^5^ cells mL^−1^ in six-well plates. Upon reaching 30% confluence, cells were infected with 10 MOI LV-Orai1 shRNA in 1 mL complete culture medium (DMEM with 10% FBS) for 8 h at 37°C. Then, the supernatant was discarded and replaced with fresh culture medium, and VSMCs cultured for an additional 96 h. The effect of Orai1 knockdown was detected using western blotting.

### Loading of Fluo-4/AM dye and confocal laser scanning microscopy for recording of intracellular levels of calcium (Ca^2+^) in coronary VSMCs

The intracellular Ca^2+^ concentration in coronary SMCs was monitored using the fluorescent dye Fluo 4-AM. Cells were loaded for 25 min in DMEM with 5 µM Fluo 4-AM, which were dissolved in dimethyl sulfoxide (DMSO; final concentration, 0.1%) and Pluronic F-127 (final concentration, <0.025%). Then, culture dishes were rinsed in standard extracellular solution containing (in mM): NaCl 132, KCl 4.8, MgCl_2_ 1.2, glucose 5, HEPES 10, and CaCl_2_ 1.8. Fluo-4 fluorescence was monitored using an inverted Confocal Laser Scanning Microscope (SP5-FCS, Leica, Mannheim, Germany). For fluorescence excitation, the 488-nm band of an argon laser was used, and the emission wavelength was 525 nm. The time-dependent change in mean fluorescence along the scanning line was used to record the intracellular Ca^2+^ concentration. The Ca^2+^ level was reported as F/F_0_ (where F_0_ is the resting fluorescence of Fluo-4).

### Quantitative real-time PCR (real-time PCR)

Total RNA was isolated from coronary SMCs or coronary artery tissue using Trizol reagent (Molecular Research Center, Cincinnati, OH, USA) according to manufacturer instructions. Total RNA (1 µg) was reverse-transcribed in a total volume of 20 µL, and real-time PCR carried out using SYBR green fluorescence. The specific primers for real-time PCR analyses were (forward and reverse, respectively): 5-HT_2A_: 5'-CAA CGG TCC ATC CAC AGA G-3' and 5'-GGG CAC CAC ATT ACA ACA AA-3'; 5-HT_2B_: 5'-TCG CCA TCC CAG TCC CTA T-3' and 5'-GCA GCC AGT GAC CCA AAG A-3'; Oria1: 5'-TGG TAG CGA TGG TGG AAG T-3' and 5'-GAC GGA GTT GAG GTT GTG G-3'; STIM1: 5'-ATG ATG CCA ATG GTG ATG TG-3' and 5'-CAT AGG TCC TCC ACG CTG ATA-3'; L-type calcium channel (Cav1.2): 5'-ACA TCT TCG TGG GTT TCG TC-3' and 5'-CACTTTGTATG GTG CTG GTT C-3'. Each real-time PCR reaction comprised 1 µL reverse transcription product, 5 µL SYBR Green PCR Master Mix, and 400 nM forward and reverse primers. Reactions were carried out with a ViiA 7 Dx Real-time PCR detection system (Applied Biosystems, Foster City, CA, USA) for 40 cycles (95°C for 25 s, 58°C for 25 s, 72°C for 25 s) after an initial 2-min incubation at 95°C. The fold change in expression of each gene was calculated using the 2^−ΔΔCt^ method with β-actin as an internal control.

### Western blotting

Coronary arteries were harvested as detailed above. Arteries were rinsed with ice-cold phosphate-buffered saline, ground with a blender, and lysed with RIPA lysis buffer containing a protease inhibitor cocktail (Merck, White House Station, NJ, USA). Protein content was quantified using a Bicinchoninic Acid kit (Thermo Scientific, Waltham, MA, USA). Protein was separated by 8% sodium dodecyl sulfate-polyacrylamide gel electrophoresis and transferred to polyvinylidene fluoride membranes (Millipore, Billerica, MA, USA). After incubation with 5% non-fat dry milk diluted with TBST (in mM: Tris–HCl 20, NaCl 150, pH 7.5, 0.1% Tween 20) at room temperature for 1 h, membranes were incubated with primary antibody against Orai1 (Alomone, Jerusalem, Israel), STIM1 (Cell Signaling Technology, Beverly, MA, USA), L-type calcium channel (Alomone), 5-HT_2A_ receptor (Millipore) and 5-HT_2B_ receptor (BD Pharmingen, Franklin Lakes, NJ, USA) at 4°C overnight. They were then incubated for 1 h with appropriate horseradish peroxidase-conjugated secondary antibodies (1∶1000 dilution; Cell Signaling Technology) at room temperature. Incubation with polyclonal rabbit Glyceraldehyde 3-phosphate dehydrogenase (GAPDH) antibody (1∶1000 dilution; Cell Signaling Technology) was done as the loading sample control. Bands were detected with Pierce ECL western blotting substrate (Thermo Scientific) and quantified with Image J software (National Institutes of Health, Bethesda, MD, USA).

### Drugs

9,11-dideoxy-11a,9a-epoxy-methanoprostaglandin F_2a_ (U46619), 5-HT, nifedipine, acetylcholine, Fluo-4, sarpogrelate, SB204741, DOI, 2-APB, thapsigargin and U73122 were purchased from Sigma–Aldrich (St. Louis, MO, USA). U-46619, thapsigargin, Fluo-4, SB204741 and nifedipine were dissolved in DMSO and the others in distilled water. Further dilution was made from a stock solution.

### Statistical analysis

All of the data are expressed as the mean ± SEM. Responses in each segment are presented as percentage of the K^+^-induced contraction. E_max_ refers to the maximal contraction induced by an agonist, expressed as a percentage of the 60 mM K+-induced response. The negative logarithm of the agonist concentration that elicited 50% contraction of E_max_ (pEC_50_) was determined by linear regression analysis using values immediately above and below the half-maximal response. Target gene mRNA levels were expressed in relation to GAPDH levels. Target protein expression was determined relative to GAPDH protein levels. For statistical analysis, a two-tailed Student's t-test or one-way analysis of variance followed by a Newman-Keuls test was used when more than two groups were compared. Individual concentration- response curves were also compared using a two-way analysis of variance followed by Bonferronic posttests. The data and statistical analysis was calculated using Sigmaplot v10.0 (Systat Software, Chicago, IL, USA). *P*<0.05 was considered to be statistically significant.

## Results

### 5-HT induced vasocontractile responses in fresh and cultured coronary arteries

The organ-culture procedure was performed as described previously[9]. First, we observed the morphological change of the cultured arteries. The width of the artery wall was not significantly changed between fresh and cultured arteries, but after organ culture the smooth muscle cells were disarranged and some nuclei of the cells turned round (Figure S1).

**Figure 1:**
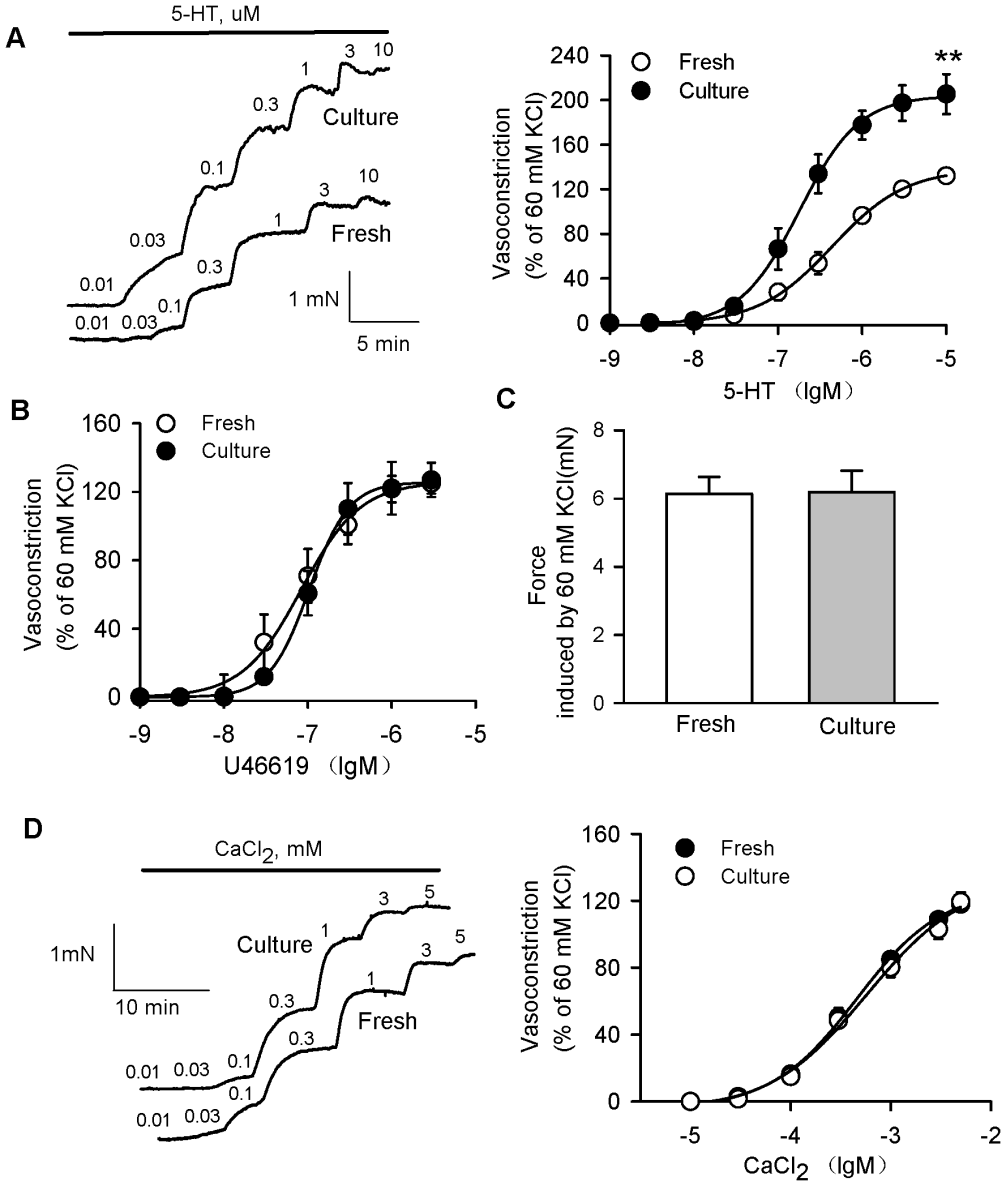
5-HT induced the upregulation of vasocontractile responses in cultured rat coronary arteries. A, Representative recording of 5-HT-evoked concentration-dependent contraction in fresh and cultured rat coronary arteries without endothelia (left panel). 5-HT induced markedly more contractions in cultured coronary arteries (full circles) than in fresh coronary arteries (open circles). B, U46619-induced contractions were comparable in fresh (open circles) and cultured coronary arteries (full circles). C, High K^+^ (60 mM)-induced contractions were comparable in fresh and cultured coronary arteries. D, Representative recording of 60 mM K^+^ solution before cumulative addition of CaCl_2_ (left panel). CaCl_2_-induced concentration-dependent contractions were not different in fresh (open circles) and cultured coronary arteries (full circles). The graph shows the mean ± SEM of 8–12 experiments on samples from different rats. **P<0.01 *vs* fresh coronary arteries.

To ascertain if contractile responses were altered in cultured rat coronary arteries, we determined vascular constrictions induced by different vasoconstrictors. The cumulative application of 5-HT produced a concentration-dependent vasoconstriction in fresh and 24 h-cultured rat coronary arteries without endothelia (Figure 1A). 5-HT induced significantly greater contractions in cultured coronary arteries than in fresh coronary arteries (E_max_: 138.3±2.38% in fresh, 204.2±2.63% in culture, *P*<0.01; EC_50_: 0.43±0.02 µM in fresh, 0.15±0.03 µM in culture, P<0.05, n = 12). U46619-(E_max_: 126.9±4.5% in fresh, and 125.9±1.5% in culture, *P*>0.05; EC_50_: 0.81±0.06 nM in fresh, 0.93±0.05 nM in culture, *P*>0.05, n = 8) and high-K^+^(60 mM)-induced contractions (tension: 6.2±0.5 mN in fresh, and 6.3±0.6 mN in culture, *P*>0.05, n = 10) were comparable in the two groups (Figures 1B and C). In addition, we also observed the 5-HT induced vasoconstriction in serum-free organ culture coronary arteries or in cultured arteries with endothelia. The increased contractility to 5-HT was comparable between serum-free cultured arteries and 10% FBS-cultured arteries (Figure S2), which excluded the possibility that FBS contributes to the augment of 5-HT induced vasoconstriction after organ culture for 24 h. The effects of 5-HT-induced contraction in the vessels with intact endothelium were not significantly different from in vessels without endothelium (Figure S3).

We further determined the contractile sensitivity to Ca^2+^. The results showed that CaCl_2_-induced contraction curves were not significantly different in the two groups (E_max_: 126.8±5.9% in fresh and 124.9±3.5% in culture, *P*>0.05; EC_50_: 0.46±0.08 mM in fresh, 0.52±0.10 mM in culture, *P*>0.05, n = 8) (Figure 1D).

### Enhanced vasoconstriction induced by 5-HT through the 5-HT_2A_ receptor in cultured coronary arteries

**Figure 2:**
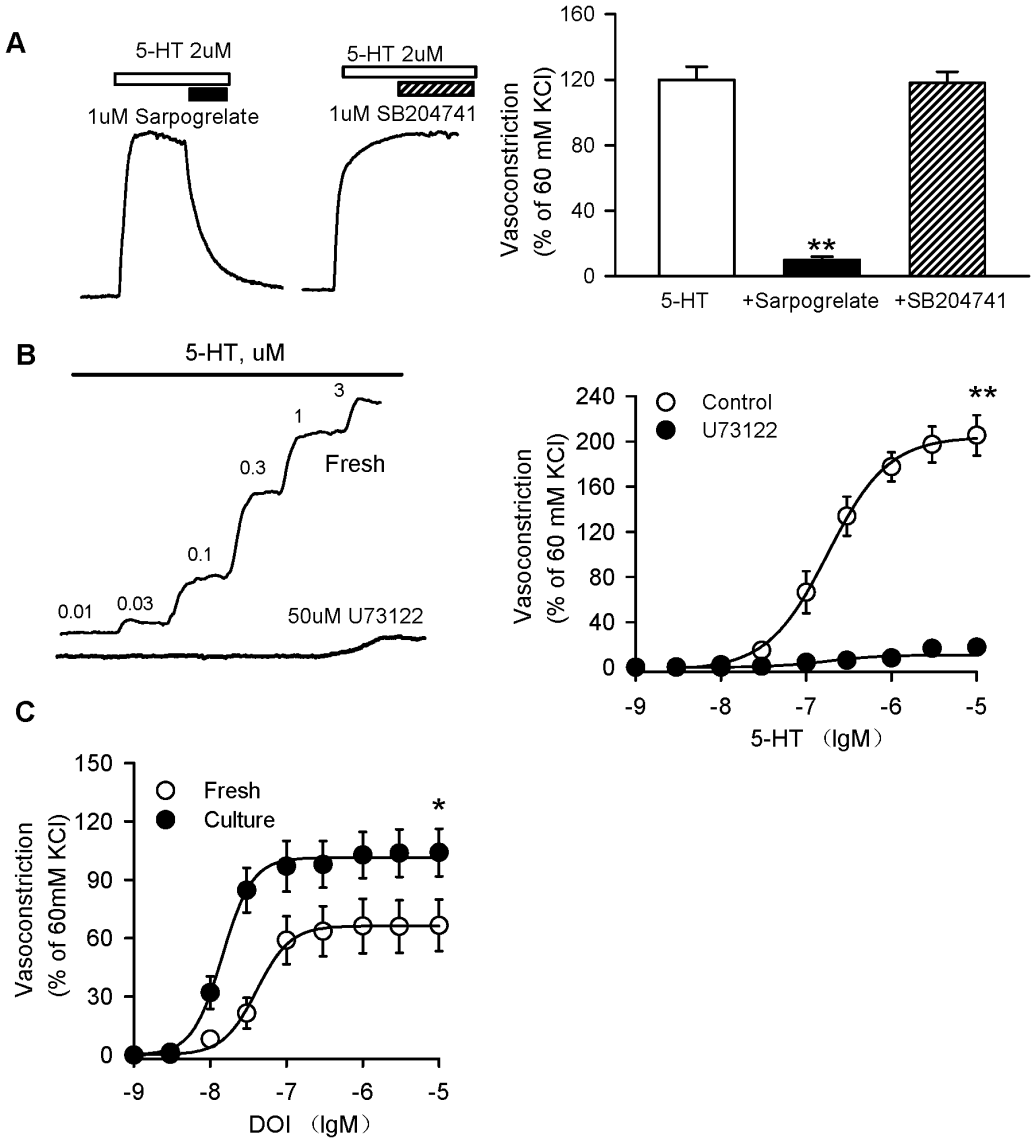
Enhanced vasoconstriction induced by 5-HT through the 5-HT_2A_ receptor in cultured coronary arteries. A, Representative recording of 5-HT-evoked contraction in cultured rat coronary arteries without endothelia, then upon addition of sarpogrelate (1 µM) or SB204741 (1 µM) (left panel). Sarpogrelate significantly reversed 5-HT-induced vasoconstriction, but SB204741 did not elicit an effect. B, Representative recording of 5-HT-evoked concentration-dependent contraction in cultured rat coronary arteries without endothelia in the presence of U73122 (50 µM). U73122 significantly prevented 5-HT-induced contraction. C, DOI (specific 5-HT2A receptor agonist) also produced an augmented contraction in cultured coronary arteries. The graph shows the mean ± SEM of 8–10 experiments on samples from different rats. *P<0.05, **P<0.01 *vs* fresh coronary arteries.

To demonstrate the involvement of subtypes of 5-HT receptors in the coronary vasoconstriction induced by 5-HT, we observed the effect of the 5-HT_2A_ receptor antagonist sarpogrelate or the 5-HT_2B_ receptor antagonist SB204741 on the vasoconstriction induced by 5-HT. Sarpogrelate at 1 µM completely reversed the vasoconstriction induced by 2 µM 5-HT. SB204741 (1 µM) did not affect the response to 5-HT (Figure 2A).

The 5-HT_2A_ receptor was functionally linked to a Gq protein whose activation stimulates PLC, so the PLC inhibitor U73122 was tested. U73122 (50 µM) significantly prevented 5-HT-induced contraction (Figure 2B). These data confirm that 5-HT stimulates the 5-HT_2A_ receptor and PLC cascade to induce coronary vasoconstriction.

The specific 5-HT_2A_ receptor agonist DOI also produced augmented contraction in cultured coronary arteries compared with fresh coronary arteries (E_max_: 66.08±1.12% in fresh, 101.5±1.3% in culture, *P*<0.05; EC_50_: 1.4±0.12 nM in fresh, 0.42±0.05 nM in culture, *P*<0.05, n = 9) (Figure 2C).

### Role of Ca^2+^ in the enhanced vasoconstriction induced by 5-HT in cultured coronary arteries

**Figure 3:**
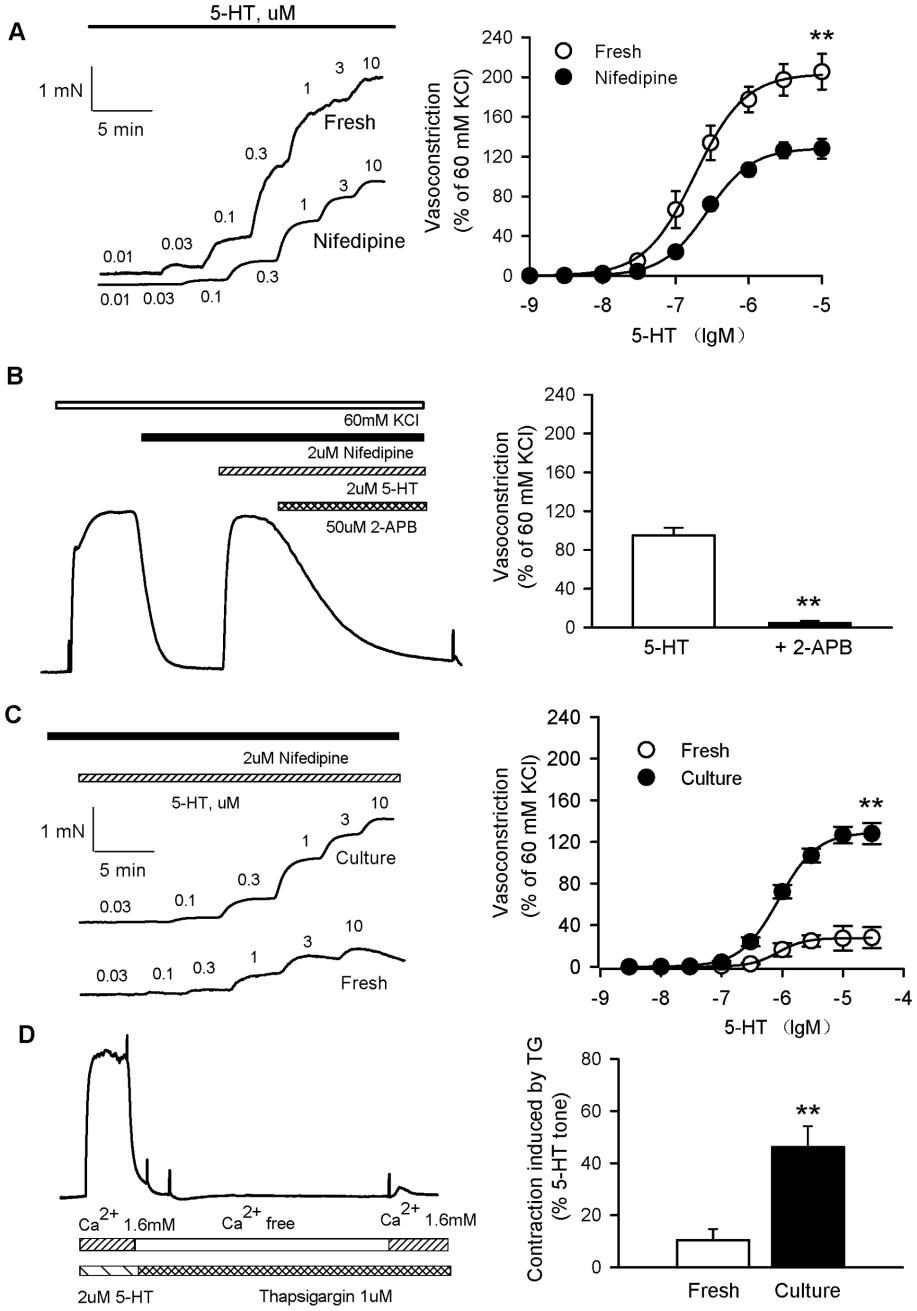
Role of Ca^2+^ in 5-HT-induced enhanced vasoconstriction in cultured coronary arteries. A, Representative recording of 5-HT-evoked concentration-dependent contraction in cultured rat coronary arteries without endothelia in the presence or absence of nifedipine (left panel). 5-HT-induced contractions were significantly suppressed by nifedipine, but inhibition was incompletely. B, High K^+^-induced contractions were first reversed with nifedipine, but subsequent addition of 5-HT could still induce contractions. 5-HT-induced contraction was blocked by the non-selective CRAC channel blocker 2-APB (50 mM) (left panel). 2-APB significantly completely inhibited the 5-HT-induced contraction in the presence of nifedipine. C, Representative recording of 5-HT-evoked concentration-dependent contraction in fresh and cultured rat coronary arteries without endothelia in the presence of nifedipine (left panel). 5-HT induced greater contractions in the presence of nifedipine in cultured coronary arteries (full circles) than in fresh coronary arteries (open circles). D, Vasoconstrictions in response to Ca^2+^ influx after depletion of intracellular Ca^2+^ stores were evaluated. Rat coronary arterial rings were stimulated with 2 µM 5-HT for 15 minutes. Then rings were washed in Ca^2+^-free EGTA buffer to deplete intracellular Ca^2+^ stores for 20 minutes. During the depletion period, arteries were treated with 1 µM thapsigargin. After Ca^2+^ depletion, intracellular Ca^2+^ stores were loaded by 1.6 mM of Ca^2+^ buffer for 15 minutes. Contractile responses during the loading period were determined (left panel). Contractions induced by thapsigargin were augmented in cultured coronary arteries (black box) compared with fresh coronary arteries (white box) during the Ca^2+^-loading period (right panel). The graph shows the mean ± SEM of 6–10 experiments on samples from different rats. **P<0.01 *vs* fresh coronary arteries.

5-HT-induced contractions were suppressed significantly by 2 µM nifedipine (specific L-type calcium blocker) in the control compared with nifedipine (E_max_: 128.7±2.2% in fresh, 200.5±3.3% in culture, *P*<0.05, n = 8), but incompletely (Figure 3A). Next, we explored the other Ca^2+^-permeable channels involved.

To explore the component that was independent of L-type Ca^2+^ channels, nifedipine first inhibited the contractions induced by high-K^+^, and subsequent addition of 5-HT still induced significant contractions in the presence of nifedipine. The 5-HT-induced contraction was blocked by 50 mM 2-APB, the non-selective Ca^2+^ release-activated Ca^2+^ (CRAC) channel blocker (Figure 3B). These results suggested that Ca^2+^ influx through L-type calcium channels and non-L-type calcium channels contributed to the contractions in the coronary artery induced by 5-HT.

In the presence of 2 µM nifedipine, the contractions in coronary arteries induced by 5-HT were greater in cultured coronary arteries (E_max_: 129.4±1.4%) than in fresh coronary arteries (E_max_: 27.6±0.3%, *P*<0.05, n = 8) (Figure 3C). This result suggested that the contractions mediated by non-L-type calcium channels were enhanced.

Furthermore, we ascertained if 5-HT-induced activation of the PLC/inositol 1,4,5-trisphosphate (IP_3_) signaling cascade would stimulate a functional store-operated Ca^2+^ entry (SOC) pathway in coronary arteries. We investigated the implication of SOC in coronary vasoconstriction. After 5-HT-induced contraction, coronary arteries were washed in Ca^2+^ free-EGTA buffer to deplete intracellular Ca^2+^ stores in the presence of thapsigargin. Then intracellular Ca^2+^ stores were loaded by 1.6 mM Ca^2+^ buffer. Contractile responses induced by thapsigargin were determined during the loading period. Thapsigargin-induced contractions were augmented in cultured coronary arteries compared with fresh coronary arteries (Figure 3D).

### Orai1 involvement in 5-HT-evoked entry of Ca^2+^ into coronary VSMCs

**Figure 4:**
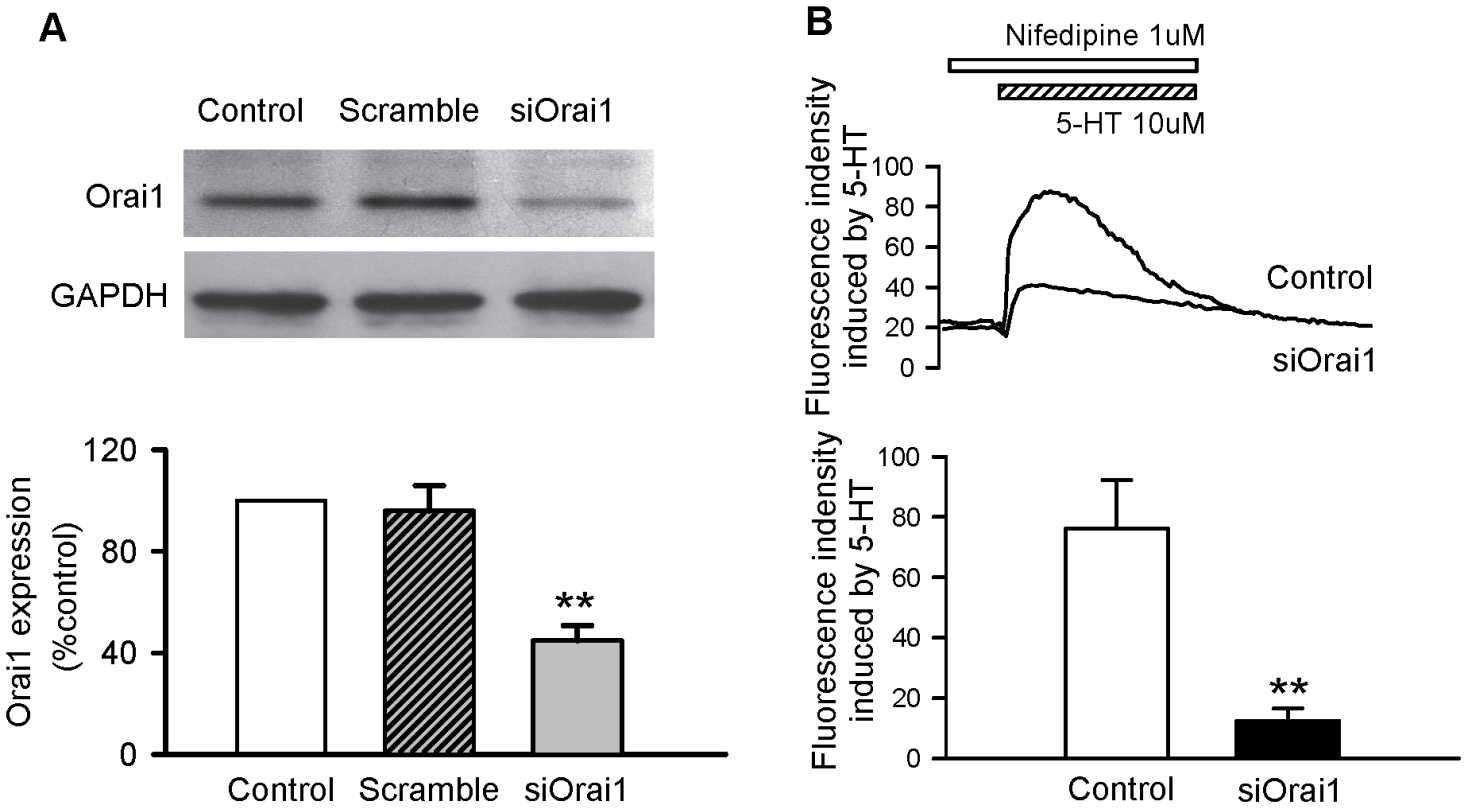
Orai1 involvement in 5-HT-evoked entry of Ca^2+^ into coronary VSMCs. A, RNA knockdown of Orai1 specifically reduced the expression of target proteins in coronary artery smooth muscle cells as assessed by western blotting. B, Orai1 downregulation significantly suppressed 5-HT-elicited Ca^2+^ influx in the presence of nifedipine 1 µM in coronary VSMCs. The graph shows the mean ± SEM of 5–8 experiments on samples from different rats. **P<0.01 *vs* control.

Once we had determined the role of SOC in 5-HT-mediated contractions, we investigated the participation of Orai1 (an essential pore subunit of the SOC channel) in 5-HT effects. We verified that RNA knockdown of Orai1 specifically reduced the expression of target proteins as assessed by western blotting (Figure 4A). Downregulation of the expression of Orai1 significantly suppressed 5-HT-elicited Ca^2+^ influx in the presence of 1 µM nifedipine in coronary VSMCs (Figure 4B).

### Upregulation of 5-HT receptor signaling in cultured coronary arteries

**Figure 5:**
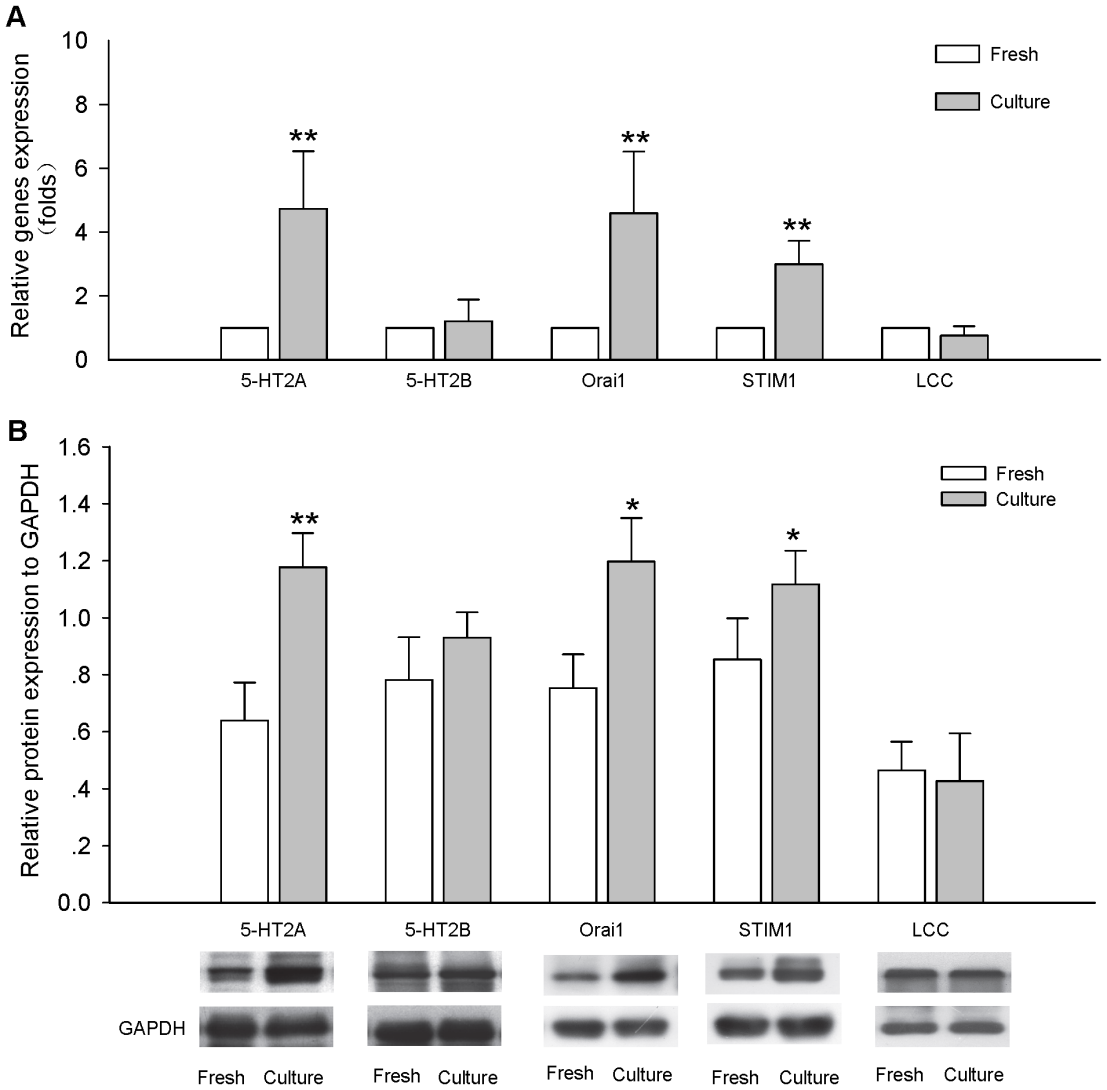
Upregulation of signaling of the 5-HT receptor is involved in contractile hyperactivity in cultured coronary arteries. A, **5-HT_2A_ receptor, Orai1, STIM1, 5-HT_2B_ receptor and L-type calcium channel mRNA expression levels in Fresh and Cultured coronary arteries.** 5-HT_2A_ receptor, Orai1, STIM1, 5-HT_2B_ receptor and L-type calcium channel were expressed relative to GAPDH levels. Expression of the 5-HT_2A_ receptor, STIM1 and Orai1 gene was augmented in cultured coronary arteries compared with fresh coronary arteries. The graph shows the means ± SEM of 4–6 experiments on samples from different rats. *P<0.05, **P<0.01 *vs* fresh coronary arteries. B, **5-HT_2A_ receptor, Orai1, STIM1, 5-HT_2B_ receptor and L-type calcium channel protein expression levels in Fresh and Cultured coronary arteries.** 5-HT_2A_ receptor, Orai1, STIM1, 5-HT_2B_ receptor and L-type calcium channel were expressed relative to GAPDH levels. Expression of the 5-HT_2A_ receptor, STIM1 and Orai1 protein was increased in cultured coronary arteries compared with fresh coronary arteries. The graph shows the means ± SEM of 4–6 experiments on samples from different rats. *P<0.05, **P<0.01 *vs* fresh coronary arteries.

The 5-HT_2A_ receptor, Orai1 and STIM1 mRNA levels were augmented in cultured coronary arteries (Figure 5A) but mRNA levels of the 5-HT_2B_ receptor and L-type calcium channel were not affected. Cultured coronary arteries exhibited the increases in protein levels of the 5-HT_2A_ receptor, Orai1 and STIM1, but those of the 5-HT_2B_ receptor and L-type calcium channel were not affected (Figure 5B). These results suggested that upregulation of expression of 5-HT_2A_ receptor and downstream signaling were involved in contractile hyperactivity in cultured coronary arteries. In addition, the protein expression of 5-HT_2A_ receptor, Orai1 and STIM1 in serum-free cultured rat coronary arteries were also examined, the results showed they were all increased (Figure S4), which indicated that organ culture procedure could induce the upregulation of 5-HT receptor signaling.

## Discussion

In the present work, the phenotypic changes in 5-HT receptors in cultured coronary arteries were examined for the first time. Our data suggested that the augmented constriction induced by 5-HT results from upregulation of the 5-HT_2A_ receptor signaling pathway in cultured coronary arteries.

First, we found that 5-HT induced an increase in vasocontractile responses in cultured rat coronary arteries. 5-HT induced coronary vasoconstriction in concentration-dependent manner. The maximal contractile response was significantly greater in cultured coronary arteries, but EC_50_ was much lower in cultured coronary arteries than fresh coronary arteries. So the potency of 5-HT increased in cultured coronary arteries. But the contraction curves induced by U46619 and CaCl_2_ showed no significant differences. These results showed that the thromboxane A2 receptor signaling pathway and vascular sensitivity to Ca^2+^ were not responsible for the enhancement of vasoconstriction after vessel culture. We further explored the mechanism of the increased vascular reactivity to 5-HT in cultured coronary arteries. We found that sarpogrelate (specific 5-HT_2A_ receptor antagonist) almost completely reversed the vasoconstriction induced by 5-HT, but that SB204741 (specific 5-HT_2B_ receptor antagonist) did not affect it in cultured rat coronary arteries. DOI (specific 5-HT_2A_ receptor agonist) also produced augmented constriction in cultured rat coronary arteries. The PLC inhibitor U73122 completely prevented 5-HT-induced vasoconstriction. These results suggest that 5-HT stimulates the 5-HT_2A_ receptor and PLC cascade to induce an increase in coronary vasoconstriction. Past researches showed that the 5-HT receptors mediating constriction in coronary arteries from different species were mostly heterogeneous, consisting of a predominant 5-HT_2_ receptor and a smaller population of 5-HT_1_ receptors[19]. The uniform population of 5-HT_2_ receptors in rat coronary arteries is unique [7], [20]. Our results also support the concept that the 5-HT_2A_ receptor signaling pathway mediates the constriction induced by 5-HT in rat coronary arteries. It has been reported that the vasoconstriction induced by 5-HT was enhanced after organ culture in rat cerebral arteries. But this response was inhibited by 5-HT_1B/1D_ receptor antagonists [15]. 5-HT stimulated the 5-HT_2A_ receptor to induce enhanced vasoconstriction after the organ culture of rat mesenteric artery ring segments removed of their endothelium [21]. Therefore, upregulation of the expression of the 5-HT_2A_ receptor contributes to the increase in contractile response to 5-HT in cultured coronary arteries.

However, previous studies have focused almost entirely on changes in the 5-HT receptor in organ cultures [9], [15]. In this experiment, we further determined changes in downstream signaling pathways after activation of the 5-HT_2A_ receptor in cultured coronary arteries. We know that the 5-HT_2A_ receptor activates PLC through G_q_ and leads to accumulation of IP_3_, diacylglycerol (DAG) and activation of protein kinase C (PKC). Increase in the cytoplasmic level of IP_3_ causes the release of Ca^2+^ from the endoplasmic reticulum (ER) [22]. Defective regulation of intracellular Ca^2+^ is a hallmark of vascular dysfunction and plays a key part in the increased vascular reactivity [23]. 5-HT elicits an increase in intracellular Ca^2+^ to induce vasoconstriction. We observed that nifedipine partially inhibited the effect of 5-HT. In the presence of nifedipine, 5-HT-induced constriction is completely inhibited by 2-APB (putative non-selective cationic channel blocker) [24]. Those results suggest that L-type and non-L-type Ca^2+^ influx channels could be involved in 5-HT_2A_ receptor-mediated constriction in rat coronary arteries. Furthermore, we found that 5-HT-induced constriction of coronary arteries through non-L-type calcium channels was enhanced in cultured coronary arteries.

Ca^2+^ influx mediated by non-L-type calcium channels was involved in SOC in VSMCs[23]. In this mechanism, the ER acts as a capacitor. SOC carries a highly Ca^2+^-selective, non-voltage-gated, inwardly rectifying current termed “CRAC current”. Activation of the 5-HT_2A_ receptor induces IP_3_-mediated release of Ca^2+^ in the ER. After depletion of Ca^2+^ from the ER, Ca^2+^ channels are activated in the plasma membrane to refill internal Ca^2+^ stores. Thapsigargin was used to inhibit Ca^2+^-ATPase in the sarcoplasmic reticulum and to promote depletion of intracellular Ca^2+^ stores[25]. We found that incubation with thapsigargin induced constriction during the Ca^2+^-loading period in both fresh and cultured coronary artery rings. Constriction was greater in cultured coronary artery rings. These results suggested that activation of CRAC channels increased in cultured coronary artery rings, thereby contributing to augmented influx of extracellular Ca^2+^. New advances in SOC research have identified STIM and Orai proteins to be key signaling molecules in several cell types, and that their interaction promotes SOC. Recently, some researches demonstrated that STIM1- and Orai1-mediated SOC and the existence of endogenous STIM1 and Orai1 proteins in rat aortic and coronary VSMCs [23], [25]. We showed that lentivirus-Orai1 shRNA infection could knockdown of Orai1 expression in coronary artery SMCs, which attenuated 5-HT-elicited Ca^2+^ influx. We suggested that, as a potential component of CRAC channels, Orai1 may contribute to the increase in intracellular Ca^2+^ induced by 5-HT in cultured coronary arteries.

Possible mechanisms associated with an abnormal function of 5-HT/PLC/Orai signaling pathways include increased activation/decreased inactivation of proteins. We observed that expression of the 5-HT_2A_ receptor, STIM1 and Orai1 were increased in cultured coronary arteries. Increased protein levels may account for the increased functional contractile responses observed after depletion of intracellular Ca^2+^. Hoel et al. reported that expression of 5-HT_1B/1D_ receptors is upregulated after organ culture in rat cerebral arteries [15], which resembles the alterations in SMC function after subarachnoidal hemorrhage [16], [17]. There is an increased level of 5-HT_2A_ receptor mRNA and of contractile 5-HT_2A_ receptors after organ culture of rat mesenteric artery ring segments in which the endothelium has been removed [21].

Our results showed that upregulation of the 5-HT_2A_ receptor and downstream signaling pathway was found to contribute to the increase in 5-HT-induced contraction, and it may be involved in the development of cardiovascular disease. Activation of 5-HT_2A_ receptor signaling elicited contraction of vascular smooth muscle to induce spasms in coronary arteries. The 5-HT_2A_ receptor appears to play a crucial part in the formation of occlusive thrombi in diseased arteries by platelet aggregation and vasoconstriction [1]. Selective 5-HT_2A_ receptor antagonists might help reduce the onset of acute coronary events and of acute coronary occlusion after the intervention [26]. Sarpogrelate, a selective 5-HT_2A_ receptor antagonist, dose-dependently inhibited the 5-HT-induced coronary spasm in porcine model [27]. Sarpogrelate increased both baseline and the maximal coronary blood flow (CBF) without changing the systemic hemodynamics in patients with coronary artery disease [28]. Sarpogrelate has been introduced clinically as a therapeutic agent for the treatment of ischemic diseases associated with thrombosis [1]. Here we showed for the first time that STIM1 and Orai1 proteins are upregulated in cultured coronary artery. Oari1 channel inhibitors also may represent a new therapeutic approach to treat coronary artery dysfunction [23].

## Supporting Information

Figure S1 **The morphological changes of the rat coronary artery after organ culture.** **A**, Light micrographs of hematoxylin and eosin sections of the fresh and 24 h-cultured rat coronary arteries (400× magnification). B, There was no change in the width of the media of rat coronary arteries after organ culture.
(TIFF)

Figure S2 **5-HT-induced contraction in serum-free cultured arteries and FBS cultured arteries.** The increased contractility to 5-HT was comparable between serum-free organ culture and serum organ culture (n = 5).
(TIF)

Figure S3 **5-HT-induced contraction in cultured rat coronary arteries with or without endothelium.** The effects of 5-HT-induced contraction in the vessels with intact endothelium were not significantly different from in vessels without endothelium (n = 5).
(TIF)

Figure S4 **Protein expression of 5-HT_2A_ receptor, Orai1 and STIM1 in serum-free cultured rat coronary arteries.** *P<0.05 *vs* fresh coronary arteries (n = 5).
(TIFF)
