# Supplementary figures and images for: Upregulation of 5-Hydroxytryptamine Receptor Signaling in Coronary Arteries after Organ Culture
Source: PLoS One. 2014 Sep 9;9(9):e107128. doi: 10.1371/journal.pone.0107128 (PMC4159325; doi:10.1371/journal.pone.0107128)

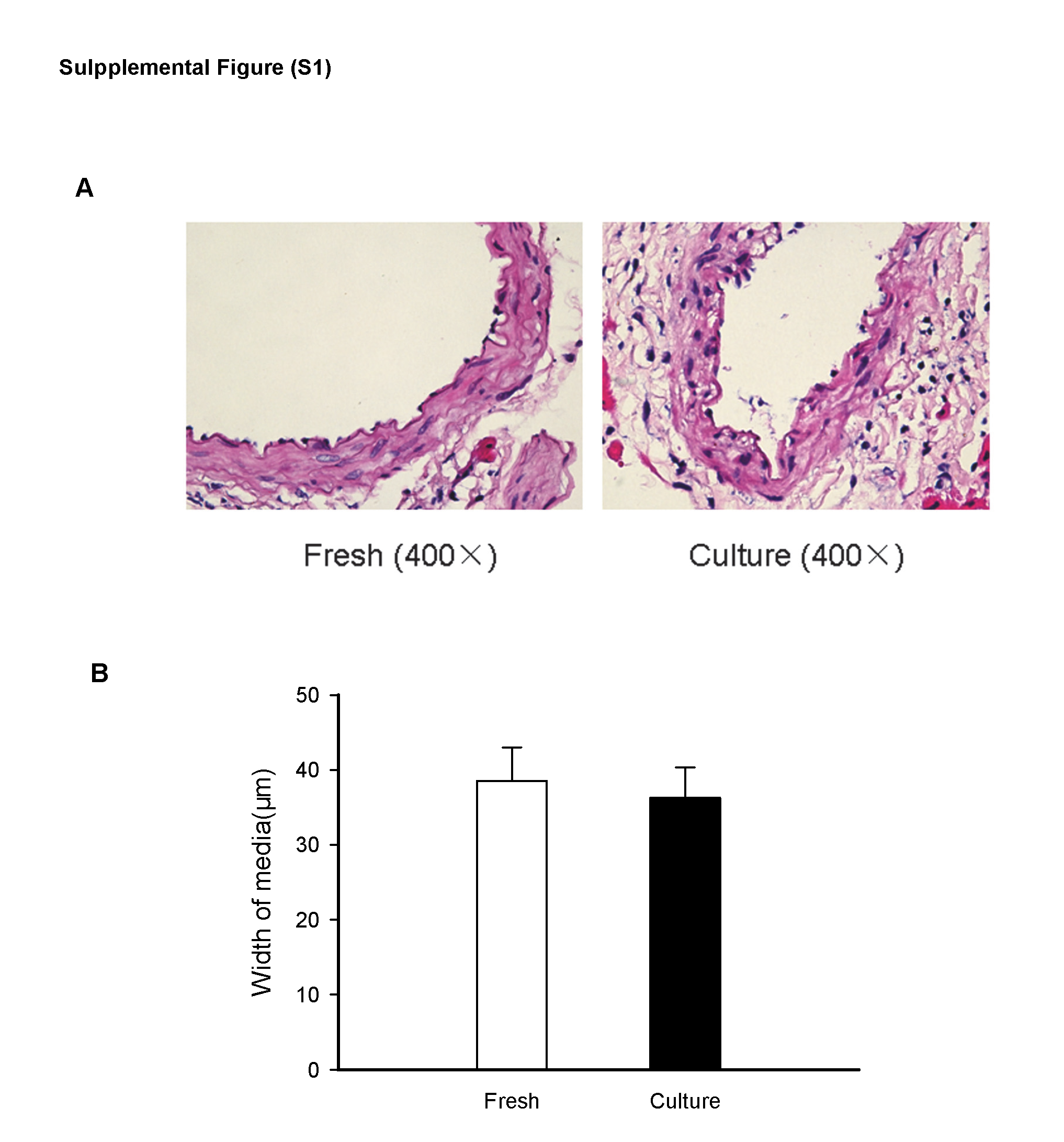

Supplement: Figure S1 — The morphological changes of the rat coronary artery after organ culture. A, Light micrographs of hematoxylin and eosin sections of the fresh and 24 h-cultured rat coronary arteries (400× magnification). B, There was no change in the width of the media of rat coronary arteries after organ culture. (TIFF) [file pone.0107128.s001.tiff]

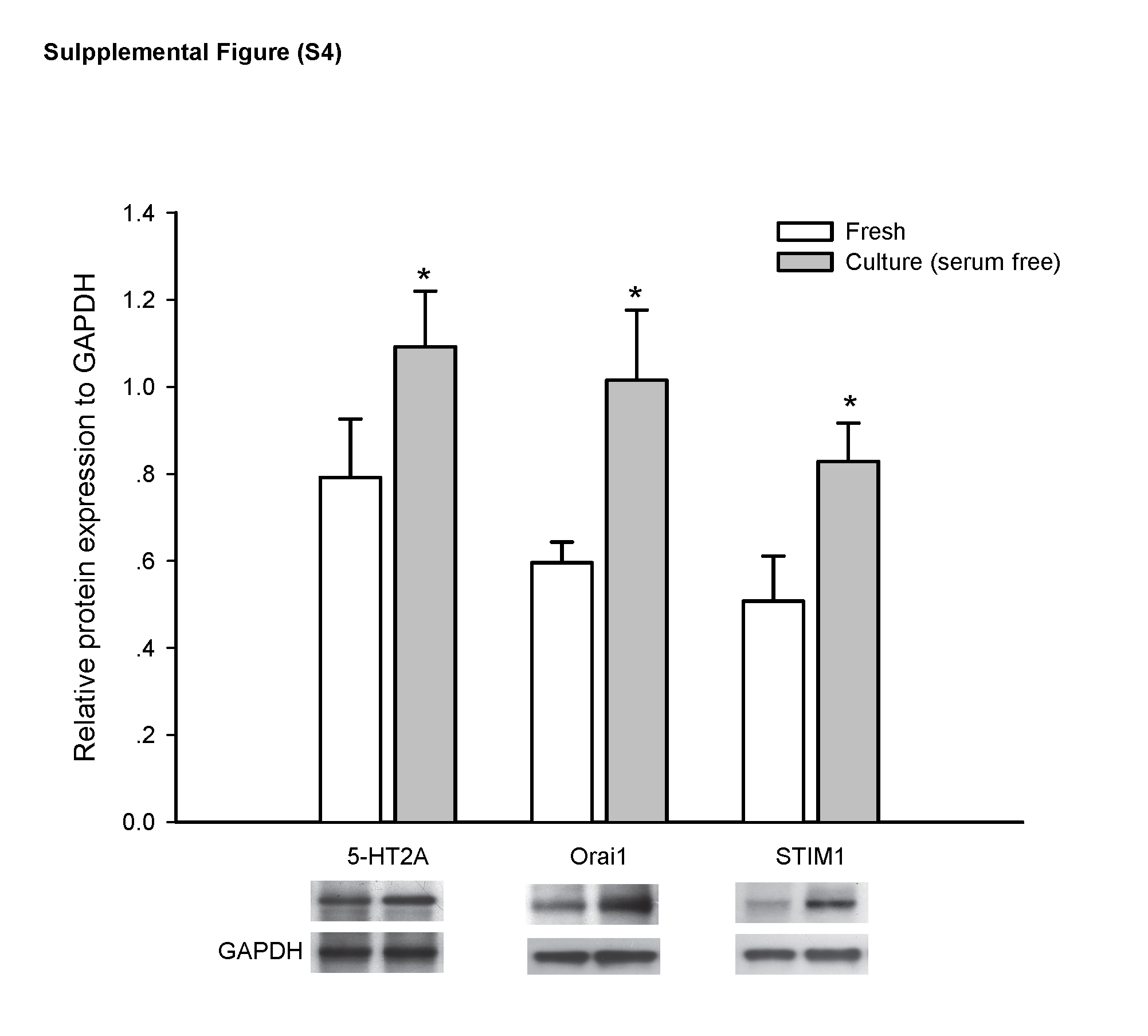

Supplement: Figure S4 — Protein expression of 5-HT2A receptor, Orai1 and STIM1 in serum-free cultured rat coronary arteries. *P<0.05 vs fresh coronary arteries (n = 5). (TIFF) [file pone.0107128.s004.tiff]
